# Developing a global occupational health and safety management system model for Japanese companies

**DOI:** 10.1002/1348-9585.12081

**Published:** 2019-08-06

**Authors:** Shigeyuki Kajiki, Koji Mori, Yuichi Kobayashi, Kou Hiraoka, Nanae Fukai, Masamichi Uehara, Nuri Purwito Adi, Shigemoto Nakanishi

**Affiliations:** ^1^ Department of Occupational Health Practice and Management Institute of Industrial Ecological Science, University of Occupational and Environmental Health Kitakyushu Japan; ^2^ Advanced Occupational Health Research and Consulting (AORC), Ltd. Tokyo Japan; ^3^ HOYA, Ltd. Tokyo Japan; ^4^ Komatsu, Ltd. Tokyo Japan; ^5^ Brother Industries, Ltd. Nagoya Japan; ^6^ Department of Community Medicine, Faculty of Medicine Universitas Indonesia Jakarta Indonesia

**Keywords:** global OHSMS, Japanese companies, performance audit, reporting system, specialized human resources

## Abstract

**Objectives:**

To develop and validate a global occupational health and safety management system (OHSMS) model for Japanese companies.

**Methods:**

In cooperation with a Japanese company, we established a research team and gathered information on occupational health and safety (OHS) practices in nine countries where the target company operated manufacturing sites. We then developed a model hypothesis via research team meeting. The model hypothesis was introduced to local factories in Indonesia and Thailand as trial sites. We evaluated the roles of the company headquarters, the implementation process, and any improvements in OHS practices at the sites. Based on the results, a global OHSMS model was formalized for global introduction.

**Results:**

The model consisted of both headquarters and site roles. These roles were well‐functioning, and OHS at the sites improved. Two issues concerning the functioning of the headquarters were identified: the need to establish a reporting system to the headquarters and the need to support the improvement of specialized human resources. By improving the model hypothesis to address these issues, the model was formalized for global introduction.

**Conclusions:**

The global OHSMS model was based on the use of methods and specialized human resources relevant to each region and their common objectives, as well as evaluation indicators based on the minimum requirements of the company headquarters. To verify the effectiveness of this model, the experiment should be extended to other countries.

## 1 INTRODUCTION

The globalization of economic activities has also seen the expansion of Japanese companies abroad. A survey of Japanese companies and their overseas subsidiaries and business activities found that there were 24 959 overseas subsidiaries of Japanese companies as of July 2017.^1^ Regarding occupational health and safety (OHS) for workers in Japan, staff in charge of OHS generally provide the necessary services in compliance with local laws and regulations. However, developing and emerging countries often lag far behind developed countries in terms of OHS training for professionals, and practices in these countries do not always keep pace with the development of laws and regulations of their home country.

Large companies in Europe and the United States typically apply global standards, which usually have stricter requirements than local regulations, to local sites, as well as complying with local OHS laws and regulations.^2^, ^3^ They often establish a unified OHS management system (OHSMS) whereby each site is required to meet common requirements, and this system is typically controlled and managed by the company's OHS department located at its headquarters.^4^ Furthermore, most OHSMSs also include international standards or company standards that reflect the laws and practices of the area in which the company's headquarters is located.^4^

In addition to considering the requirements in the location of the company's headquarters, which is involved in personnel allocation and investment decision‐making through capital relationships, as part of risk management and corporate social responsibility strategies, Japanese companies, as well as Western companies, need to improve their OHS standards at their subsidiaries, regardless of location.^4^, ^5^

When considering OHS practices based on Japanese regulations, it should be noted that Japanese OHSMSs are based on minimizing requirements and assume that the resources and personnel are adjusted to the actual situation in each region as much as possible to achieve the same objective. Thus, we call our system a “global OHSMS for Japanese companies.”

In developing a global OHSMS for Japanese companies, it is necessary to gather information on the OHS environments in the target countries and regions where overseas subsidiaries are located and to establish a system that enables both the involvement of the company headquarters and the autonomous efforts of local sites. Thus, we developed an “Information Collection Check Sheet for OHSMSs at Overseas Plants” as a tool for efficient information gathering,^6^ and investigated the actual conditions regarding, for example, OHS regulations and human resource development in a number of countries.^7^, ^8^, ^9^, ^10^

In this study, we developed a global OHSMS model for Japanese companies and confirmed its validity via cooperation with the headquarters of a global Japanese manufacturing company that produces construction equipment.

## 2 METHOD

### 2.1 Research team

We established a research team that consisted of two experienced occupational physicians (OPs: SK, KM) working at a Japanese branch of a US‐based global company, two chief OPs (YK, MS) with Japanese‐based global enterprises, and the chief OP (SN) and two in‐house OPs (KH, NF) from the target company, which is described below.

### 2.2 Target company

The target company is a manufacturer of construction and mining equipment that has 12 production sites in Japan and 31 overseas sites. Of the overseas sites, 18 are located in five Asian countries, including China, seven are located in five European countries, including Russia, five are located in the United States, and one is located in Brazil. The company has approximately 60 000 employees worldwide, of which 60% are non‐Japanese employees working at the overseas sites.

The company produces a diverse range of products, and total sales in the 2016 fiscal year were approximately 1.8 trillion yen. The possible health hazards for workers include noise, heat, dust, organic solvents, and bad posture. Company management displayed a clear willingness to promote both OHS and OHS investment in all workplaces, including its overseas sites.

### 2.3 Model development process

The model was developed in four steps: (a) information gathering; (b) establishing the model hypothesis and developing evaluation indicators; (c) introducing the model hypothesis at test sites and evaluating the roles of the company headquarters; and (d) improvement of the model hypothesis based on the results of the pilot implementation and completion of the global OHSMS model.

#### 2.3.1 Information gathering

Because most European sites exist in countries that are members of the European Union, the OHS requirements are considered to be similar across these sites. Therefore, to better understand the overall picture of the similarities and differences among the overseas sites, we surveyed nine countries from various continents, including Japan.

**Figure 1:**
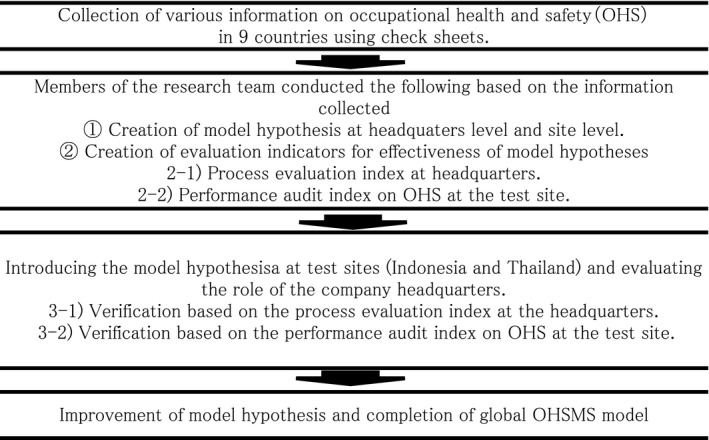
This figure shows the flow of this research for about 6 years

To conduct the survey, we visited each of the nine countries following a literature and Internet search and gathered information using the “Information Collection Check Sheet for OHSMS at Overseas Plants”.^6^ Members of the research team visited the Japanese Embassy, local administrative agencies such as the Ministry of Health, Labor and Welfare, ISO (International Organization for Standardization) accreditation bodies, and institutions that train OHS experts, such as universities, in each country. We conducted interviews with representatives in each location that lasted for about 2–3 hours. If sufficient information was not obtained during the initial visit, we returned to the site as many times as necessary. A flow chart of the survey procedure is shown in Figure 1.

#### 2.3.2 Establishing the model hypothesis and developing evaluation indicators

We conducted a meeting of the research team to establish a model hypothesis based on the information obtained. Following a brainstorming session with members of the research team, we created the categories at headquarters level and site level respectively. To establish a hypothesis based on a global OHSMS perspective, we clarified that it will be implemented at the headquarters and sites on the premise of utilizing the basic policy and OHSMS.

Then, we created evaluation indicators for headquarters and test sites to confirm the validity of the model hypothesis. Evaluation indicators were also developed based on the ideas presented by the research team members during the discussion. The evaluation indicators made it possible to evaluate the level of improvement in detail and to describe what kind of reach (state) each criterion is specifically. Headquarters adopted process evaluation and test sites created performance audit on OHS.

#### 2.3.3 Introducing the model hypothesis at test sites and evaluating the roles of the company headquarters

We conducted a pilot implementation of the global OHSMS for Japanese companies in Indonesia and Thailand based on the model hypothesis. While the sites in these countries had already introduced a number of OHS initiatives, there was considerable room for improvement, mainly in the field of occupational health. For example, neither the established OHS practices based on laws and regulations nor the risk management system addressing hazardous factors not covered by laws and regulations were satisfactory. The training system in these two countries was relatively clear, and it was easy to obtain specialized human resources from major universities and administrative agencies. Furthermore, these countries were selected because local management was committed to introducing the OHSMS.

Based on the hypothesis, we gained an understanding of the OHS conditions at the sites in these countries through an interview and field patrol with local health and safety personnel. Then, the research team presented proposals to the site management teams regarding the measures necessary to address OHS issues that had been identified. In cases where the site personnel required assistance from OHS experts, we introduced local experts to management, and also recommended the use of OHS experts when it was necessary to provide education and training for workers.

Then, we evaluated the effects of our intervention after a period of time. The research team examined the functions and roles of the company headquarters and evaluated after developing efforts to test sites. The evaluation result of the company headquarters was determined by the members of the research team. Evaluation of the local sites was based on previously determined evaluation indicators (performance audit consists of evaluation items and criteria). Members of the survey team discussed the audit results with the top management and safety and health managers of the target factory and discussed until the audit team and the audited organization were satisfied.

#### 2.3.4 Improvement of model hypothesis based on the results of the pilot implementation and completion of the global OHSMS model

We reviewed the results of the performance audit and evaluation following the pilot implementation. Then, we reviewed the model hypothesis, discussed any necessary improvements, and completed the global OHSMS model.

## 3 RESULTS

### 3.1 Information gathering

The results of the survey of OHS systems in nine countries, including Japan, are as follows.

#### 3.1.1 Laws on OHSMS and status of specialized human resources

Regarding presence of laws and guidelines on OHSMS, Japan, Thailand, and China welcomed the introduction of an OHSMS.

Regarding professionals mainly responsible for OHS activities, OPs were active in Japan, Indonesia, Germany, and Brazil. Safety Officers are active in Thailand and China. In the United Kingdom, family physicians, and in the United States, Safety Professionals and Industrial Hygienists have been the main activities.

Regarding legal requirements concerning the appointment and utilization of expert personnel in OHS, Japan, Indonesia, Germany, and Brazil are all legally required to appoint OPs. Japan, Thailand, China, and Germany have legal obligations regarding the appointment of safety managers or safety officers.

Regarding status of training specialized human resources on OHS, OPs in Japan and Indonesia were able to obtain qualifications by receiving more than 50 hours (Japan) and 56 hours (Indonesia) of training. Different levels of SOs were developed in Thailand and China. OP specialists were trained in Germany, the United Kingdom, the United States, and Brazil. Although there is no requirement to employ specialist staff such as OPs and safety officers, the responsibilities of businesses are stipulated, and specialist personnel are employed in the United Kingdom and the United States (see Table 1).

**Table 1:** Laws on OHSMS and status of specialized human resources of nine countries using the information collection check sheet

No	Country name	Presence of laws and guidelines on OHSMS	Professionals mainly responsible for OHS activities	Legal requirements concerning the appointment and utilization of expert personnel in OHS	Status of training specialized human resources on OHS
1	Japan	Yes	OPs	The obligation to appoint an OP and the establishment of SM, HM, etc. are stipulated by laws and regulations at business establishments of a certain size or more.	OP qualifications can be obtained by taking education of 50 hours or more based on the standards of laws and ordinances.
2	Indonesia	No	OPs	There is an obligation to provide therapeutic medical services by an OP who directly employed in workplaces of a certain size or more.	OP qualifications can be acquired by taking education of 56 hours or more based on the standards of laws and ordinances. Certified specialist OPs are cultivated at several domestic universities.
3	Thailand	No There is a management system standard called TIS 18001, but there is no obligation to certify.	SOs	OHS activities are structured to focus mainly on SOs. There are five levels of SO. Specialized OPs perform special health examination.	The number of certified OPs being trained is not large. SO has been trained in 86 facilities as of February 2015.
4	China	No Safety production standardization exists as a framework for promoting OHS activities, and applicable business sites need to comply with these requirements.	SOs	Establishment standards for SOs are determined. Only institutions that have qualified doctors who are certified by the government can conduct special health checkups.	SOs can be in charge of graduation above the vocational school or those who received a certain training. There are no certified OPs, but public health doctors (medical departments) are being trained at universities throughout the country.
5	Myanmar	No	None	None	None
6	Germany	No	OPs	There is an obligation to appoint an OP, a SO. Those persons in charge can be an employee or an external expert.	OPs and SOs are trained.
7	United Kingdom	No	Family Doctor	There is no obligation to appoint an OP. There are cases where external consultants are utilized to satisfy the requirements specified by laws and ordinances.	Family doctor is playing the role of OP. There are qualified OPs.
8	United State of America	No	SPs His	There is no obligation to appoint a person in charge of OHS experts.	CSP (Certified SP) and CIH (Certified IH) are cultivated. Specialized OPs are cultivated at several domestic universities, and there is a specialist medical system certified by academic societies.
9	Brazil	No	OPs External experts	There is an obligation to appoint safety engineers, occupational health nurses, OPs.	Training of specialist OP is being conducted.

Abbreviations: HM, hygiene manager; IH, industrial hygienist.; OHS, occupational health and safety; OHSMS, occupational health and safety management system; OP, occupational physician; SM:safety manager; SO, safety officer; SP, safety professional.

#### 3.1.2 Status of major programs related to occupational health

Regarding presence of law of risk assessment for harmful factors, Japan, Germany, the United Kingdom and Brazil have to conduct and personal exposure monitoring for hazardous work is conducted in the United States.

Regarding evaluation of health effects by hazardous work, employers are obliged to carry out evaluation of health effects by hazardous work in all countries except for Myanmar. There are those in which health checks and laboratory standards are regulated by law (eg, China) and those where they are selected by specialized personnel (eg, Indonesia, Thailand, and Germany).

Regarding fit for work programs, all countries except for Myanmar have laws and regulations prescribing pre‐deployment health checks for workers engaged in hazardous work. The United States is only required when a worker returns to work after injury.

Regarding management of personal information, personal health information is only shared between the workers and medical professionals, and only health‐related information that is relevant to the workers’ employment is conveyed to employers. In Myanmar, OHS legislation is not well‐developed, and there is no clear provision for any of the above practices (see Table 2).

**Table 2:** Status of major programs related to Occupational Health of nine countries using the information collection check sheet

No	Country name	Presence of Law of RA for harmful factors	Evaluation of health effects by hazardous work	Fit for work program	Management of personal information
1	Japan	Yes There are RA and chemical substance RA guidelines.	There is an obligation for SME. For each harmful factor, inspection items are stipulated by law.	There is an obligation of GME (before deployment, regular, special worker). There are guidelines on support for returning to mental health disabled people. Efforts are under way to support work and treatment compatibility at the same time.	The results of GME are obliged to be preserved by the operator for 5 years. Personal information is provided to business operators after processing by OPs and other.
2	Indonesia	No	There is an obligation for SME. Details of inspection items are not stipulated by laws and regulations.	There is a judgment classification of GME. More concrete judgment criteria and post correspondence according to health condition are defined for each employment classification.	No regulations concerning Personal information are stipulated.
3	Thailand	No	There is an obligation for SME at the time of employment and change of workplace. The details of inspection items are not stipulated in laws and ordinances.	If there is a finding on the result of SME by workers, arrange for the workers to receive medical treatment immediately and investigate the cause for prevention.	The employer shall record the results of all SME of workers engaged in hazardous work in the personal medical examination notebook.
4	China	No	SME must be carried out for workers engaged in hazardous work at the time of employment and at the time of changing workplaces. Inspection items are stipulated for each harmful factor by law. Standards of the executing agency are stipulated.	If a company finds a health hazard resulting from that job, it is necessary to relocate the workplace of that worker properly.	It is necessary to prepare and preserve health management records including data on health aspects concerning individuals such as worker's work history, past records that touched on occupational disease harm.
5	Myanmar	No	None	None	None
6	Germany	Yes There is an obligation to conduct RA.	There is an obligation to conduct SME.	There is an obligation to conduct GME(before deployment, regular, special worker). A list of OI and WRD exists and OP advises.	Individual medical information and health information can be confirmed only by OPs.
7	United Kingdom	Yes There is an obligation to conduct RA. Management itself places emphasis on self‐management of business operators.	There is an obligation to conduct SME. Details of inspection items are not stipulated by law.	Fit note, which is the application form for official leave of compensation, is used at the time of reinstatement from sick leave. Workers first consult with their superiors and employers, and consideration is often given to employment on that basis.	The results of SME are to be managed by the business operator. Results of health examination will be notified to individual workers as well.
8	United State of America	No Using the results of PEM, voluntary RA and measures based on the results are required.	There is an obligation to conduct SME for special work obtained by law.	At the time of reinstatement after occupational accidents or labor diseases, it is required to prepare a workplace where businesses can arrange by referring to the opinion written on the doctor's medical certificate at the time of reinstatement.	The results of SME are managed by the company (in‐house personnel in charge). The results of GME (voluntary implementation) are managed only by individual workers and are not notified to business operators.
9	Brazil	Yes The risk of hazardous work inside the workplace is identified by experts in occupational health and safety outside the company.	There is an obligation to conduct SME.	OPs conduct assessment of job aptitude and state opinions to companies.	Only workers and OPs can view the results of SME and GME.

Abbreviations: GME, general medical examination; MH, mental health; OI, occupational injury; OP, occupational physician; PEM, personal exposure monitoring; PI, personal information; RA, risk assessment; SME, specific medical examination; WRD, work‐related disease.

### 3.2 Establishing the model hypothesis and developing evaluation indicators

#### 3.2.1 Model hypothesis

We assumed a model consisting of two levels of practices at the company headquarters and at each manufacturing site. In the headquarters, the following eight practices were implemented at the headquarters level: “formulation and dissemination of health and safety policies by the CEO (Chief Executive Officer),” “establishment of a global safety and health conference,” “determination of OHSMS standards,” “formulation and notification of global standards,” “formulation and notification of performance audit standards,” “training of auditors and conduct performance audit,” “support for securing and fostering appropriate human resources at each site,” and “technical support in the case of a shortage of specialized resources.”

Of these, the global standards issued by the headquarters of Japanese companies included those used to promote the autonomous activities of the organization and to acquire budget funding. To facilitate verification of the introduction of global standards, we developed the following global standards: “risk assessment,” “chemical substance management,” and “facilities and personal protective equipment standards.”

The components of an OHSMS that were developed and applied at each site are as follows: “formulation of the basic policy,” “specialized human resources in OHS or utilization of external resources,” “companies’ global standards and regulations compliance,” “promotion of autonomous activities,” and “internal audit and continuous improvement.”

The OHSMS at each site was based on the Occupational Health and Safety Assessment Series (OHSAS) 18001 in consideration of an integrated review with ISO 14001 and the possibility of future global integrated authentication. At the time of the development of the model hypothesis, ISO 45001 (which is similar to OHSAS 18001) was expected to become an ISO standard in the near future. Therefore, it was decided to replace OHSAS 18001 with ISO 45001 when it was made official.

#### 3.2.2 Evaluation indicators

To evaluate the global OHSMSs of Japanese companies, we decided to use process evaluations at the headquarters level and performance audits at the test sites. Process evaluation items at the headquarters level were “expression of company‐wide basic policy on health and safety,” “development and dissemination of company‐wide global standards required to be implemented at each site,” “opportunities for information sharing among staffs in charge of OHS at each site,” and “establishment of indicators for performance audits conducted from the headquarters standpoint and training of auditors.”

The performance audits at the test sites were conducted over 2 days by four researchers at each site. The audit team proposed the scoring system in relation to the performance audit evaluation (evaluation items and criteria) to top management and the OHS manager at the test site, and the scores were determined based on mutual agreement. Evaluation items at the test site were based on the following 12 items after discussion among research team members: (1) introduction of management systems, (2) appointment of personnel in charge of safety and health, organizational positioning, and job authority, (3) competency of personnel in charge of safety and health, (4) description in the management system of specialized resources (people/organizations) in relation to safety and health, (5) compliance, (6) risk assessment, (7) risk reduction measures, (8) evaluation of health of workers exposed to harmful factors, (9) evaluation of job aptitude and suitability for employment (fit for work), (10) management of personal information, (11) emergency preparedness in relation to OHS functions, and (12) prevention of recurrence of work‐related illnesses. Each evaluation criteria was scored on a 10‐point Likert scale ranging from “0: Procedure (criterion) does not exist” to “9: Procedure (criterion) is executed reliably and continuously until it reaches a level that is a model both inside and outside the company” (see Table 3).

**Table 3:** Trends in evaluation indicators before and after interventions in Indonesia and Thailand (test sites)

Evaluation item	Site in Indonesia		Site in Thailand	
	Aug.2013	Jun.2016	Jun.2013	Aug.2016
(1) Introduction of management system	2	5	2	3
(2) Appointment of the persons in charge of safety and health, organizational positioning, job authority	3	5	3	4
(3) Competency of person in charge of safety and health,	3	5	3	5
(4) Description in the management system of specialized resources (people/organization) for safety and health	2	5	2	3
(5) Compliance	4	5	4	5
(6) Risk assessment	3	4	0	3
(7) Risk reduction measures	2	3	0	3
(8) Evaluation of health effects of workers exposed to harmful factors	2	4	2	4
(9) Evaluation of job aptitude and consideration of employment (fit for work)	0	4	0	3
(10) Management of personal information	4	4	0	5
(11) Labor during crisis management function that takes safety and health into consideration	4	5	3	5
(12)Prevention of recurrence after occurrence of work‐related illness	4	4	2	4

Definition of numbers: 0: Procedure (criteria) does not exist. 1: There is a procedure (criteria) but it has not been introduced. 2: There are procedures (criteria) and some have been introduced. 3: There is a procedure (criteria) but there is a significant issue that needs to be addressed before it can be introduced. 4: There is a procedure (criteria) but there is a minor issue that needs to be addressed before it can be introduced. 5: Procedure (criteria) is clearly executed. 6: Evaluation of effectiveness of procedure (criteria) is continually performed (there is a mechanism). 7: Procedures (criteria) are executed (reliably and continually) and have achieved consistent results. 8: The procedure (criteria) has been (reliably and continually) executed and has achieved high results. 9: Procedure (criteria) is (reliably and continually) executed, and it is at a level whereby it is a model inside and outside the company.

### 3.3 Introducing the model hypothesis at test sites and evaluating the roles of the company headquarters

#### 3.3.1 Test site in Indonesia

##### Problems before introduction of model hypothesis

We conducted the first performance audit at the Indonesian test site in August 2013. The audit showed that this site complied with laws and regulations, that an OHSMS had not been introduced, and that there were problems regarding a number of occupational health practices including risk assessment, chemical substance management, and health checks.

##### Specific activities to improve (September 2013 to May 2016)

After being introduced to global policies and draft global standards, professional staff from the company's headquarters provided education and training for workers. We also assisted site personnel in obtaining advice from local OHS experts.

##### Improvements after implementation and evaluation

We conducted a second performance audit in June 2016, and the results from before and after the implementation of the model hypothesis were compared. OHSAS 18001 certification, which is an international standard for OHS management, was acquired in January 2014, and practices from the Japanese‐based sites such as “Safety Dojo,” KY (danger prediction), and 5S (Sorting, Setting‐in‐Order, Shining, Standardizing, and Sustaining the Discipline) activities were implemented at the Indonesian site. The test site entered into a consultancy contract with the Department of community medicine, University of Indonesia, and under the guidance of an occupational medicine expert, hazard identification was performed and a hazard list (eg, the creation of a noise map) was compiled. Then, risk assessment was undertaken based on the hazard list. The relevant aspects of the health‐check process for workers engaged in hazardous work were also reviewed. Selection and education in the use of personal protective equipment was carried out and a professional OP was hired.

As a result of these efforts, the following six evaluation items improved by 2 points or more. (1) introduction of management systems, (2) appointment of personnel in charge of safety and health, organizational positioning, and job authority, (3) competency of personnel in charge of safety and health, (4) description in the management system of specialized resources (people/organizations) in relation to safety and health, (8) evaluation of health of workers exposed to harmful factors and (9) evaluation of job aptitude and suitability for employment (fit for work).These efforts resulted in changes in the site's scores, as shown in Table 3.

#### 3.3.2 Test site in Thailand

##### Problems before introduction of model hypothesis

We conducted the first performance audit at the site in Thailand in June 2013. The audit showed that safety and health management was being carried out under ISO 14001, and that there was compliance with the relevant laws and regulations. However, we found some problems regarding the development of risk management processes based on risk assessment and occupational health practices such as chemical substance management and health checks. A safety officer was hired and provided with appropriate training, including training in occupational health.

##### Specific activities to improve (July 2013 to July 2016)

We did not seek support for the expert personnel, but we did explain the global policy and the global standard plan, and the specialist staff at the company headquarters continued to implement risk assessment training. Because the safety officer who was hired did not have sufficient knowledge and experience, we recommended external training.

##### Improvements after implementation and evaluation

We conducted a second performance audit in August 2016. OHSAS 18001 certification was obtained in April 2015, and existing practices from the Japanese‐based factories were implemented. Furthermore, a health and safety officer was placed in the manufacturing department, and existing workplace hazards were identified and a hazard list was created. Health checks for workers engaged in hazardous work were reviewed by the new safety officer. The health‐check procedure was amended based on recommendations from an external OP. The in‐house safety officer worked with external labor health agencies and began to use the results of the health checks to determine fit for work.

As a result of these efforts, the following eight evaluation items improved by 2 points or more. (1) Competency of personnel in charge of safety and health, (2) risk assessment, (3) risk reduction measures, (4) evaluation of health of workers exposed to harmful factors, (5) evaluation of job aptitude and suitability for employment (fit for work), (6) management of personal information, (7) emergency preparedness in relation to OHS functions and (8) prevention of recurrence of work‐related illnesses. These efforts resulted in changes in the site's scores, as shown in Table 3.

#### 3.3.3 Evaluation of the company headquarters

The Japanese headquarters published global safety and health policies from April 2011 to August 2016. These outlined the company's behavioral standards, and global safety and health policies were transmitted to domestic and overseas business sites. It was decided that annual global health and safety meetings would be held and that OHSAS 18001 (now ISO 45001) or equivalent management system standards would be introduced. Performance audit related to OHS were also formulated, and initiatives to systematically audit a number of domestic and overseas business sites each year in accordance with the appropriate standards were implemented. Regarding the training of auditors, staff with relevant knowledge and experience were selected from within the company, and on‐the‐job training was provided. Efforts were made to secure and nurture specialized talent in each country to fulfill contracts between local institutions and overseas affiliates. If difficulties arose in dealings with a specific country, an OHS expert from Japan was dispatched to the site. Regarding global standards, guidelines for risk assessment, personal protective equipment, and chemical substance management were formulated and communicated.

The eight practices included in the model hypothesis at the company headquarters level were discussed and examined by the research team, who evaluated the degree to which each item had been achieved. Discussion continued until a unanimous decision was reached in relation to each item. As a result, the following nine practices were identified as the roles of headquarters. To establish and disseminate basic policy on health and safety by CEO. To establish global safety and health conference. To determine OHSMS standard. To formulate and notify global standards for OHS. To formulate and notify performance audit standards. To train auditors and conduct performance audits. To evaluate the competence and expertise of employed professionals and provide support to secure and develop specialized human resources as necessary. To provide technical support in the case of shortage of specialized resources. To clarify the reporting route to the headquarters of activities related to OHS at the site.

### 3.4 Improvement of model hypothesis based on the results of the pilot implementation and completion of the global OHSMS model

As a result of the pilot implementation, the effectiveness of the model hypothesis was generally confirmed, with two issues being identified by the research team. First, it was deemed necessary to clarify the reporting mechanism to enable the company headquarters to better understand and compare the situation at each site. Second, the local professionals who were appointed lacked sufficient knowledge and experience in relation to the hygiene and health sectors.

**Figure 2:**
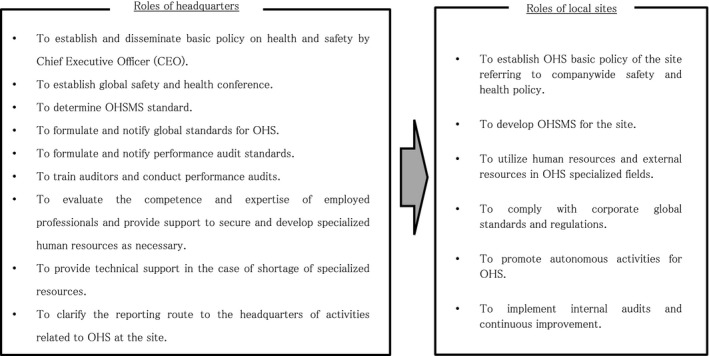
This figure shows the global OHSMS model for Japanese companies newly developed by this research group

As mentioned previously, safety awareness processes that are unique to Japanese companies, such as “Safety Dojo” and KY, were also introduced to overseas sites. However, when the research team first developed the model hypothesis and evaluation indicators, we did not see the need to evaluate these activities. Therefore, they were excluded from the evaluation in the pilot implementation. After these issues were addressed, the global OHSMS model for Japanese companies was completed, as shown in Figure 2.

The global OHSMS model included the following features: the inclusion of OHSAS 18001 (now ISO 45001), which is an international management system standard, the announcement of the policy by the CEO, the establishment of a coordination system between the company headquarters and local sites, the use of local expertise to collaborate with universities and other institutions, education and training of personnel, compliance with laws and regulations, preparation of in‐house global OHS standards, and performance audits. After obtaining approval from management at the headquarters in Japan, the company finalized the global OHSMS model and decided to implement it globally.

## 4 DISCUSSION

We developed a global OHSMS model in four steps to enable a common standard of OHS practice at all of a company's sites, including overseas sites.

In the first step, based on a previous study,^6^ it was necessary to gather detailed information on OHS activities, which are conducted in accordance with each country's laws and regulations and the available human resources in terms of OHS professionals. Therefore, the research team gathered information in advance using the Internet, visited each country selected in the study sample, and gathered information on site by conducting interviews with various stakeholders.^7^, ^8^, ^9^

In the second step, it was necessary to develop and verify a model for an OHS system that could be used at overseas sites. Therefore, the research team discussed the information that had been compiled and developed a model hypothesis based on the results. In addition, evaluation indicators were developed. Furthermore, we emphasized the promotion of autonomous practice and human resource development at each site.

In the third step, it was necessary to select overseas sites to test the model hypothesis and verify the effects, and to undertake pilot implementations. In addition, it was necessary to enumerate the efforts that were necessary on the part of the Japanese headquarters. Therefore, based on the information relating to each country gathered during the first step,^7^, ^8^, ^9^ and with the cooperation of the management of the company, two countries, Indonesia and Thailand, were selected for pilot implementations.

In the fourth step, based on the results obtained from the pilot implementations, the research team verified the validity of the model hypothesis and evaluation indicators. Finally, we improved the model based on our findings following the above process.

### 4.1 Pilot implementation in two countries (Indonesia and Thailand)

Based on the theory assumed at the research team meeting, we conducted pilot implementation in Indonesia and Thailand. In the results using the evaluation indicator, the six and eight items have significantly improved in Indonesia and Thailand, respectively.

The common weakness was observed in occupational health programs, such as evaluation of health of workers exposed to harmful factors and evaluation of job aptitude and suitability for employment (fit for work) before implementation. One of the success factors at the pilot sites was evaluating the existing OHS expertise and improving it by obtaining advice external experts or enhancing skills of internal experts with training.^5^ In addition, the implementation of risk assessment and risk reduction was not sufficiently implemented at the sites. Therefore, when introducing the global OHSMS, it was considered important to strengthen risk assessment education,^5^ provide company‐wide guidelines,^4^ and secure experts who completed comprehensive OHS training.^5^

### 4.2 Headquarters functions and roles promoting the global OHSMS model

In order to support the introduction of the global OHS model, the headquarters played several significant roles.^5^ Among them, OHS policies by CEO and the OHS global conference, which brings together representatives from around the world, show the direction of the company and it is presumed that it was effective for information sharing and network construction.^4^, ^5^ It is also speculated that the common safety and health system was established at sites around the world by recommendation of obtaining the certification of the international standard of OHSMS (ISO45001^11^).

Performance audit that evaluates OHS activities with common items clarifies the characteristics and issues of each business site, and it offers opportunities for improvement of OHS activities.^3^, ^12^ The process and report of the performance audit should be accepted not only by the headquarters of Japan but also by the management of local business sites. The performance audit team was composed mainly of members of the research team who had auditor experience in the past.^12^ Since audits require conversations in English, they may need to have certain language skills as well as OHS knowledge. Since the performance audit was extremely important for the operation of the global OHSMS model and verification of the effects,^5^, ^12^ it should be considered to secure competent auditors by training inside resources contracting with outside ones.

### 4.3 Reporting system to the health and safety department in headquarters regarding performance audit results

The evaluation of the performance audit identified two issues regarding the model hypothesis. In relation to the company headquarters’ reporting system, each site currently reports to the manufacturing department, but not to the health and safety department. To enable an understanding of the OHS situation at each site and an evaluation of the effectiveness of the global standards, it is necessary to build a regular business reporting line to the company headquarters’ health and safety department.

### 4.4 Need for utilization of occupational health experts

To promote autonomous practices based on the minimum standards contained in the model hypothesis, it is essential to use specialized OHS resources.^2^ While such specialized resources are used in Europe and the United States,^13^, ^14^ most Asian countries only have access to limited resources. The legal obligations in relation to the appointment of specialized staff also vary widely among countries.

Of the countries in which the model was introduced, Indonesia is required to appoint a doctor who has undergone a short period of training.^7^ This legal requirement already existed at the time of the pilot implementation. However, the level of expertise was not considered sufficient, and was greatly improved by using an OP who undertook systematic training to obtain a professional qualification.

Meanwhile, in Thailand, the placement of a safety officer with an undergraduate degree from a faculty of public health is mandatory,^8^ and this was already in place at the time of the pilot implementation. However, this requirement did not provide the officer with sufficient experience, and therefore the existing occupational health programs need further improvement.

Thus, in this model, it is desirable to select experts who are familiar with the local situation (eg, OPs and certified experts) in each country. This means that this need will be secured as a special resource in the occupational health field, and its effect will be clarified by recognizing it as a requirement. In developing countries, where it is difficult to obtain specialized resources, it is necessary to consider support from company headquarters and/or neighboring countries.

### 4.5 Necessity of evaluation of practices to increase workers’ awareness

Some practices were not subject to evaluation in the performance audits. These included Safety Dojo and KY^15^ activities. In Japan, major safety practices must comply with various laws and regulations, and until risk assessment becomes mandatory,^16^, ^17^ measures must be developed to increase safety awareness. Thus, many overseas sites are making significant efforts in areas other than risk management. It is necessary to promote risk assessment, prioritize risks in the workplace, and strategically promote risk reduction. In addition, voluntary efforts to raise awareness of the health and safety of workers are also important.^18^ A performance audit evaluates such efforts, and therefore improvements are essential, and are also a feature of the proposed management system.^3^

### 4.6 Necessity of management leadership

Managing a global OHSMS requires leadership from top management^19^ and this initiative was implemented with strong support from top management. They recognized that OHS issues at their overseas sites involved numerous risky practices. Thus, recognition and cooperation from top management at the company headquarters is indispensable for successful implementation at the local sites.

### 4.7 Characteristics of a global OHSMS model for Japanese companies

Our global OHSMS model has the following features: (a) it introduces ISO 45001 as the framework for the OHSMS^11^; (b) the standard issued by the company headquarters is the minimum standard, including the basic global policy for OHS; (c) it uses the most appropriate professionals in the area; and (d) it includes a performance audit to confirm the effectiveness of the system and to provide opportunities for improvement.

To enable Japanese companies to establish consistently sound OHS practices at all sites, including overseas sites, we propose to introduce a management system that serves as an overall framework. ISO 45001 is a global standard,^11^ and each country has appropriate resources such as a certification body. Furthermore, from the viewpoint of the company, it is advantageous if it possesses the possibility of integrated authentication with other ISO systems (eg, ISO 14001 and ISO 9001). However, by only introducing ISO 45001, we do not believe that OHS practices at overseas sites will improve to the level required. The introduction of ISO 45001 by Japanese companies is considered to be merely a “necessary condition” for the development of global OHS activities, including at overseas sites.^4^, ^5^

Regarding the items issued by the company headquarters, these are limited to understanding the situation at each site and enabling comparisons, and by the need to secure OHS budget allocations. In countries where detailed requirements are already set out in various laws and ordinance,^17^ there may be discrepancies and duplications between the company headquarters’ standards and local laws and customs. For example, if health checks are mandatory in Japan, inconsistencies will arise in Western countries where health‐check results are not used to determine a worker's ability to perform certain tasks because of privacy concerns. In addition, if measurement of the working environment is mandated, duplication occurs (such as requiring both practices in the country of management) based on personal exposure measurements. Thus, the company headquarters needs to act consistently in accordance with the global OHSMS model.

Under the proposed model, it is necessary to conduct performance audits on a regular basis, for example, every three years. This will ensure that conformity with standards is monitored, as well as the degree of conformity. Continuous improvement can also be evaluated. Therefore, it is essential that quality is maintained via performance audits conducted by internally trained auditors. If a global OHSMS model for Japanese companies is introduced and the understanding of the necessary internal standards by experts and staff in charge of OHS at each site is improved, then it will also be possible for them to act as auditors.

### 4.8 Limitations

This model was established in relation to a specific company, and its validity was only confirmed in two emerging countries. Therefore, it is necessary to verify its effectiveness via full implementation throughout all of the company's overseas sites and through its application in other industries.

## 5 CONCLUSION

The validity of our global OHSMS model was confirmed by the fact that company headquarters’ roles functioned properly under the model, while OHS practices at the test sites were improved as a result of the intervention. We will further evaluate the effectiveness of the model by introducing it to all of the major manufacturing sites of the target company and by extending it to other companies in the near future.

## DISCLOSURE

*Approval of the research protocol*: N/A. *Informed consent*: N/A. *Registry and the registration no. of the study/trial*: N/A. *Animal studies*: N/A. *Conflict of interest*: The first author, Shigeyuki Kajiki, and one co‐author, Yuichi Kobayashi, are health and safety consultants at Komatsu Ltd. Another co‐author, Shigemoto Nakanishi, is an OP at Komatsu Ltd. The other co‐authors have no conflicts of interest to disclose.

## AUTHOR CONTRIBUTIONS

SK, KM, YK, and MU conceived the ideas, KH and NF collected the data, NP analysed the data and advised the revision of idea, and KM and SN led the writing.
